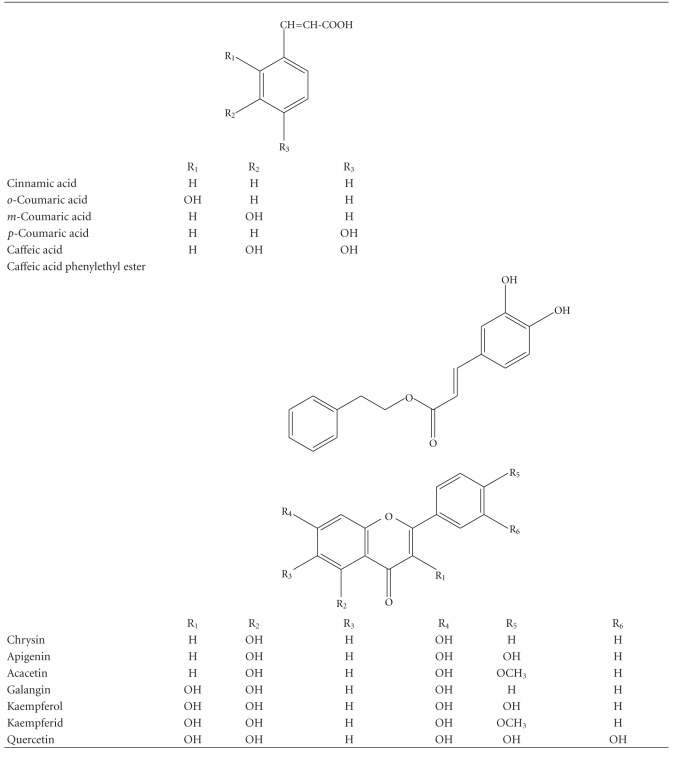# Ethanolic Extract of Propolis Augments TRAIL-Induced Apoptotic Death in Prostate Cancer Cells

**DOI:** 10.1093/ecam/nep180

**Published:** 2011-06-07

**Authors:** Ewelina Szliszka, Zenon P. Czuba, Joanna Bronikowska, Anna Mertas, Andrzej Paradysz, Wojciech Krol

**Affiliations:** ^1^Chair and Department of Microbiology and Immunology, Jordana 19, 41808 Zabrze, Poland; ^2^Chair and Department of Urology, 3-go Maja 13, 41 800 Zabrze, Medical University of Silesia in Katowice, Poland

## Abstract

Prostate cancer is a commonly diagnosed cancer in men. The ethanolic extract of propolis (EEP) and its phenolic compounds possess immunomodulatory, chemopreventive and antitumor effects. Tumor necrosis factor-related apoptosis-inducing ligand (TRAIL/APO2L) is a naturally occurring anticancer agent that preferentially induces apoptosis in cancer cells and is not toxic to normal cells. We examined the cytotoxic and apoptotic effects of EEP and phenolic compounds isolated from propolis in combination with TRAIL on two prostate cancer cell lines, hormone-sensitivity LNCaP and hormone-refractory DU145. The cytotoxicity was evaluated by MTT and LDH assays. The apoptosis was determined using flow cytometry with annexin V-FITC/propidium iodide. The prostate cancer cell lines were proved to be resistant to TRAIL-induced apoptosis. Our study demonstrated that EEP and its components significantly sensitize to TRAIL-induced death in prostate cancer cells. The percentage of the apoptotic cells after cotreatment with 50 **μ**g mL^−1^ EEP and 100 ng mL^−1^ TRAIL increased to 74.9 ± 0.7% for LNCaP and 57.4 ± 0.7% for DU145 cells. The strongest cytotoxic effect on LNCaP cells was exhibited by apigenin, kaempferid, galangin and caffeic acid phenylethyl ester (CAPE) in combination with TRAIL (53.51 ± 0.68–66.06 ± 0.62% death cells). In this work, we showed that EEP markedly augmented TRAIL-mediated apoptosis in prostate cancer cells and suggested the significant role of propolis in chemoprevention of prostate cancer.

## 1. Introduction

Prostate cancer is a commonly diagnosed cancer in men, and it is the second leading cause of death due to cancer in men in the European Union and in the USA. The rate of prostate cancer among all new cancer cases has been estimated at 12% in the EU and 29% in the USA. The molecular mechanisms responsible for the initiation and progression of prostate cancer have not been elucidated, and the only established risk factors for this disease include age, ethnic group, diet and hereditary susceptibility [1]. Prostate cancer behavior is mostly unpredictable; however, its longer time of progression to malignancy and metastasis provides broader possibilities for its managements, including the suitability for chemopreventive intervention. Chemoprevention is a rapidly growing area of uro-oncology, which focuses on prevention of prostate cancer using naturally occurring or synthetic agents [2, 3]. Many plant and animal extracts show various biological activities, such as immunopotentiating and antitumor properties [4–6].

Propolis (bee glue) is a resinous hive product collected by honey bees from many plant sources. Propolis usually contains a variety of different chemical compounds, including phenolic acids or their esters, flavonoids (flavones, flavanones, flavonols, dihydroflavonols and chalcones), terpenes, aromatic aldehydes and alcohols, fatty acids, stilbenes and *β*-steroids [7, 8]. Propolis cannot be used in its crude form, and so it must be purified by extraction to remove the inert material and preserve the polyphenolic fraction. The ethanolic extract of propolis (EEP) has attracted researchers' interest in the last decades because of its biological and pharmacological properties, such as immunomodulatory and anticancer effects [9–11]. Several mechanisms contribute to the overall cancer preventive and antitumor properties of propolis and its phenolic components. Further study demonstrated that flavonoids, phenolic acids, as well as EEP inhibit the cancer cells proliferation and tumor growth, induce cell-cycle arrest and apoptosis [10–14].

The target of much research has been on discovery of natural and synthetic compounds that can be used in the prevention of cancer. Epidemiological and preclinical evidence suggest that polyphenols isolated from propolis possess cancer chemopreventive properties [12]. Due to the fact that propolis is a rich source of plant phenolics and polyphenolics, it can be used as a dietary supplement in prostate cancer prevention.

The role of host immune functions has become increasingly important in our understanding of the mechanisms involved in cancer prevention. EEP stimulated nonspecific immunity, activated humoral immunity, and enhanced cell-mediated immunity [10, 15]. The increase of the host immune defence by propolis against tumor cells suggests that immunomodulatory effects of EEP may be involved in cancer chemoprevention.

Tumor necrosis factor-related apoptosis inducing ligand (TRAIL), a member of TNF superfamily, selectively induces apoptosis in cancer cells with no toxicity against normal tissues. Soluble, or expressed on lymphocytes T, macrophages and NK cells molecules, TRAIL plays an important role in immune surveillance and defence mechanisms against tumor cells. The cytotoxic effector functions of those immune cells are important for enabling the immune system to cope efficiently with malignancy. TRAIL induces programed death in various cancer cells through its interaction with the death receptor TRAIL-R1 and/or TRAIL-R2 [16].

However, some tumor cells are resistant to TRAIL-mediated cytotoxicity. The decreased expression of death receptors TRAIL-R1 and TRAIL-R2 or increased expression of antiapoptotic protein in cancer cells are involved in TRAIL-resistance. We and others have shown that TRAIL-resistant prostate cancer cells can be sensitized by chemotherapeutic agents, ionizing radiation, or dietary polyphenols [17–19].

In this work, we investigated the apoptotic and/or cytotoxic effect of EEP and some of its phenolic derivatives in combination with TRAIL on prostate cancer cells. We showed for the first time that EEP sensitizes prostate cancer cells to TRAIL-induced apoptosis. Our results indicated that EEP markedly augments TRAIL-mediated apoptosis in hormone-sensitivity LNCaP and hormone-refractory DU145 prostate cancer cells. The TRAIL-mediated cytotoxic and apoptotic pathways may be a target of the chemopreventive agents in prostate cancer cells, and the overcome of TRAIL-resistance by propolis and its phenolic components may be one of the mechanisms responsible for their cancer preventive effects.

## 2. Methods

### 2.1. Propolis Sample and EEP

Propolis was collected manually from beehives located in southern Poland (The Carpathians, Nowy Sacz region) and kept desiccated pending its processing. It was extracted in 95% (v/v) ethyl alcohol, in a hermetically closed glass vessel for 4 days at 37°C, under occasional shaking. The ethanolic extract was then filtered through a Whatman filter paper no 4 and evaporated in a rotary evaporator, under reduced pressure at 60°C. The same collection and extraction procedures were used throughout all our laboratory studies [9]. EEP was dissolved in DMSO (50 mg mL^−1^), and the final concentration of DMSO in the culture medium was controlled at 0.1% (v/v).

### 2.2. Flavonoids and Phenolic Acids

Propolis samples from various geographical areas contain different compounds. The major active components of propolis from Poland are flavonoids and phenolic acids or their esters [7]. All tested compounds were detected in our sample of EEP as described previously [9].  Table 1 presents the structures of compounds found in the tested sample of EEP. Chrysin, apigenin, acacetin, galangin, kaempferol, kaempferid, quercetin, cinnanic acid, *o*-coumaric acid, *m*-coumaric acid, *p*-coumaric acid, caffeic acid and caffeic acid phenylethyl ester (CAPE) were purchased from Carl Roth GmbH (Karlsruhe, Germany) and Sigma Chemical Company (St Louis, MO, USA). The reagents were dissolved in DMSO (flavonoids and phenolic acids—50 mM) and the final concentration of DMSO in the culture medium was controlled at 0.1% (v/v). The final concentration of flavonoids and phenolic acids was 50 *μ*M (chrysin, 12.7 *μ*g mL^−1^; apigenin, 13.5 *μ*g mL^−1^; acacetin, 14.2 *μ*g mL^−1^; galangin, 13.5 *μ*g mL^−1^; kaempferol, 14.3 *μ*g mL^−1^; kaempferid, 15.0 *μ*g mL^−1^; quercetin, 15.1 *μ*g mL^−1^; cinnanic acid, 7.4 *μ*g mL^−1^; *o*-coumaric acid, 8.2 *μ*g mL^−1^; *m*-coumaric acid, 8.2 *μ*g mL^−1^; *p*-coumaric acid, 8.2 *μ*g mL^−1^; caffeic acid, 9.0 *μ*g mL^−1^; CAPE, 14.2 *μ*g mL^−1^). 

### 2.3. TRAIL

Recombinant human TRAIL was purchased from PeproTech (Rocky Hill, NJ, USA).

### 2.4. Prostate Cancer Cells Culture

The experiments were performed on two human prostate cancer cell lines: hormone-sensitivity LNCaP cells and hormone-refractory DU145 cells (DSMZ—German Collection of Microorganisms and Cell Cultures, Braunschweig, Germany). The cells were grown in monolayer cultures in RPMI 1640 medium containing 10% fetal bovine serum, 4 mM l-glutamine, 100 U mL^−1^ penicillin and 100 *μ*g mL^−1^ streptomycin and incubated at 37°C in atmosphere containing 5% CO_2_ [19]. Reagents for cells culture were purchased from PAA The Cell Culture Company (Pasching, Austria).

### 2.5. Cytotoxicity Assay

The cytotoxicity was measured by 3-[4,5-dimethylthiazol-2-yl]-2,5 diphenyltetrazolium (MTT) assay as described [19]. The LNCaP cells (2 × 10^5^ mL^−1^) and DU145 (1 × 10^5^ mL^−1^) were seeded 48–24 h before the experiments onto a 96-well plate. Various combinations of EEP (5–50 ng mL^−1^) with or without TRAIL (50–200 ng mL^−1^), flavonoids (50 *μ*M) with or without TRAIL (100 ng mL^−1^), and phenolic acids (50 *μ*M) with or without TRAIL (100 ng mL^−1^) were added to the cells, and, after 48 h, the medium was removed, and 20 *μ*L of a MTT solution prepared at 5 mg mL^−1^ (Sigma Chemical Company, MO, USA) were added to each well for 4 h. The resulting crystals were dissolved in DMSO. Controls included native cells and medium alone. The spectrophotometric absorbance of each well was measured using a microplate reader (ELx 800, Bio-Tek Instruments, Winooski, VT, USA) at 550 nm. The percent cytotoxicity was calculated by the formula: percent cytotoxicity (cell death) = (1 − [absorbance of experimental wells/absorbance of control wells]) × 100%.

### 2.6. Lactate Dehydrogenase Release Assay

Lactate dehydrogenase (LDH) is a stable cytosolic enzyme that is released upon membrane damage in necrotic cells. LDH activity was measured using a commercial cytotoxicity assay kit (Roche Diagnostics GmbH, Mannheim, Germany), in which LDH released in culture supernatants is measured with a coupled enzymatic assay, resulting in conversion of a tetrazolium salt into red formazan product. The prostate cancer cells were treated with EEP in various concentrations (5–50 ng mL^−1^) alone and in combination with TRAIL (50–200 ng mL^−1^), phenolic compounds (50 *μ*M) alone and in combination with TRAIL (100 ng mL^−1^) for the indicated period of time. The sample solution (supernatant) was removed, and LDH released from cells was measured in culture medium. The maximal release was obtained after treating control cells with 1% Triton X-100 (Sigma Chemical Company, St. Louis, MO) for 10 min at room temperature [19]. The necrotic percentage was expressed using the formula (sample value/maximal release) × 100%.

### 2.7. Determination of Apoptotic Cell Death by Annexin V-FITC Staining

Prostate cancer cell line LNCaP (2 × 10^5^ mL^−1^) and DU145 (1 × 10^5^ mL^−1^) were seeded in 24-well plates for 24–48 h and then exposed to EEP and/or TRAIL for 48 h. After 48-h incubation, cancer cells were washed twice with PBS and resuspended in 1 mL of binding buffer. Five hundred microliters of cell suspension were then incubated with 5 *μ*L of annexin V-FITC and 10 *μ*L of propidium iodide (PI) for 10 min at room temperature in the dark. Annexin V assay was performed using the Apoptotest-FITC Kit (Dako, Glostrup, Denmark). The population of annexin V-positive cells was evaluated by flow cytometry (BD FACScan, Becton Dickinson Immnunocytometry Systems, San Jose, CA, USA) [20].

### 2.8. Statistical Analysis

The results are expressed as mean ± SD obtained from three separate experiments. The experimental means were compared to the means of untreated prostate cancer cells harvested parallelly, and the data were polled for replicate experiments. Statistical significance was evaluated using one- and multiple-way ANOVA or Kruskal–Wallis test followed by the Levene *post hoc* test. *P*-values < .05 were considered significant.

## 3. Results

### 3.1. Induction of Cytotoxicity and Apoptosis by Studied Agents on Prostate Cancer Cells

#### 3.1.1. EEP

**Figure 1:**
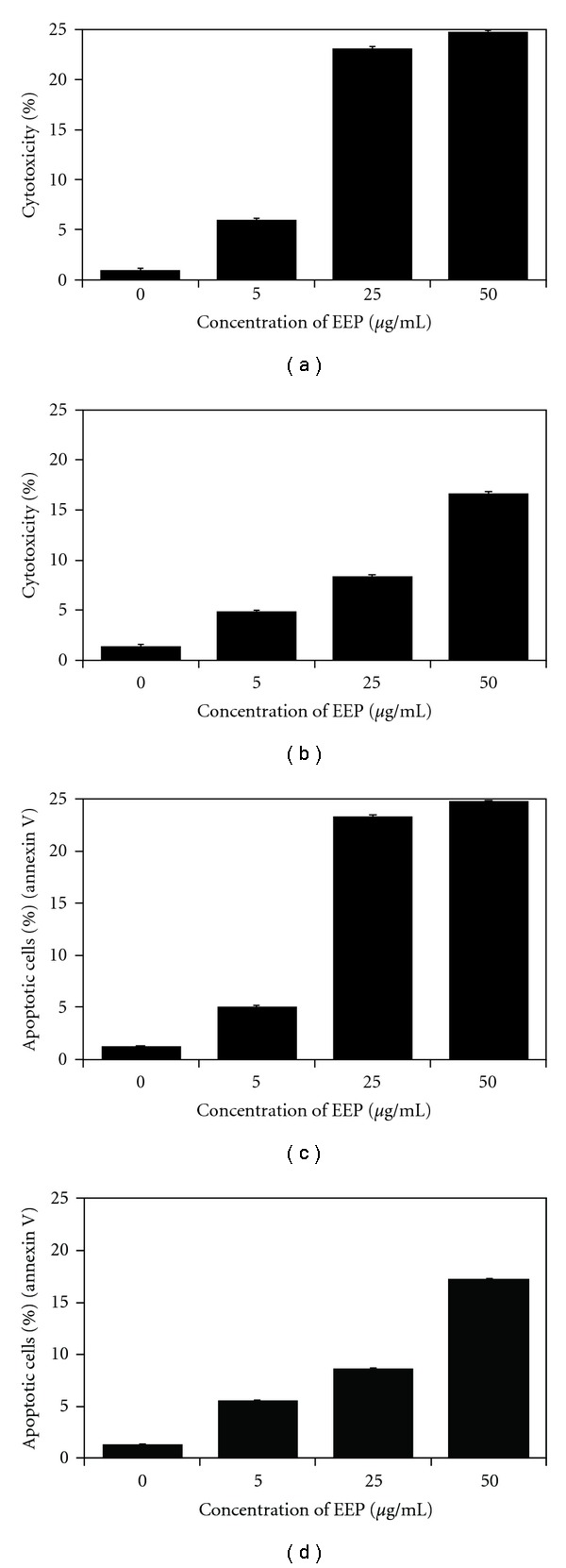
Induction of cytotoxicity and apoptosis by EEP in prostate cancer cells. The cells were incubated for 48 h with EEP at concentrations of 0–50 *μ*g mL^−1^. Cytotoxic activity of EEP in prostate cancer cells: (a) LNCaP and (b) DU145. The percentage of cell deaths was measured by MTT cytotoxicity assay. EEP induced apoptosis in prostate cancer cells: (c) LNCaP and (d) DU145. Detection of apoptotic cell death by annexin V-FITC staining using flow cytometry. The values represent mean ± SD of three independent experiments performed in quadruplicate (*n* = 12). All differences are statistically significant in relation to control (*P* < .05).

EEP inhibited growth and induced apoptosis in prostate cancer cells in a dose-dependent manner. The cytotoxic and apoptotic effects of EEP on hormone-sensitivity LNCaP and hormone-refractory DU145 prostate cancer cells are given in Figure 1. The cells were incubated with 5–50 *μ*g mL^−1^ EEP for 48 h. The rate of cytotoxicity upon treatment cancer cells with 5, 25 and 50 *μ*g mL^−1^ EEP was 5.96 ± 0.61, 23.08 ± 0.78 and 24.83 ± 0.59% for LNCaP cells and 4.75 ± 0.67, 8.20 ± 1.12 and 16.63 ± 0.77% for DU145 when compared with untreated control, respectively. The annexin V assay revealed apoptotic prostate cancer cells exposed to EEP. We showed that EEP at the concentrations of 5–50 *μ*g mL^−1^ induced 4.95 ± 0.54, 23.28 ± 0.54 and 24.66 ± 0.72% apoptosis in LNCaP cells and 5.44 ± 0.45, 8.52 ± 0.48 and 17.09 ± 0.55% apoptosis in DU145 cells. 

#### 3.1.2. TRAIL

**Figure 2:**
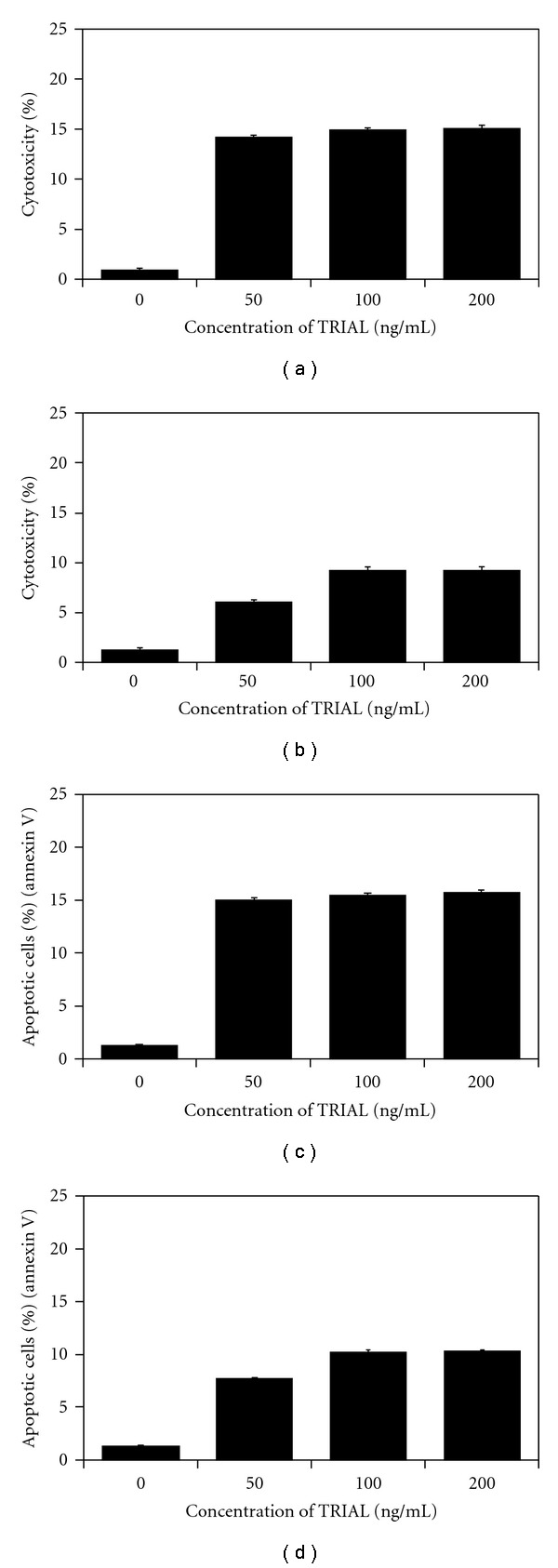
Induction of cytotoxicity and apoptosis by TRAIL in prostate cancer cells. The cells were incubated for 48 h with TRAIL at concentrations of 0–200 ng mL^−1^. Cytotoxic activity of TRAIL in prostate cancer cells: (a) LNCaP and (b) DU145. The percentage of cell deaths was measured by MTT cytotoxicity assay. TRAIL induced apoptosis in prostate cancer cells: (c) LNCaP and (d) DU145. Detection of apoptotic cell death by annexin V-FITC staining using flow cytometry. The values represent mean ± SD of three independent experiments performed in quadruplicate (*n* = 12). All differences are statistically significant in relation to control (*P* < .05).

TRAIL induced cytotoxic and apoptotic effects in a dose-dependent manner (Figure 2). We first measured the cytotoxic activity of TRAIL after 48-h incubation on prostate cancer cells. The cytotoxicity of TRAIL at the concentration of 100 ng mL^−1^ on LNCaP cells was 15.03 ± 0.50%, and on DU145 cells 9.25 ± 0.86%. TRAIL increased the percentage of apoptotic cells. For example, a 48-h exposure to 100 ng mL^−1^ TRAIL induced apoptosis of 15.46 ± 0.55% LNCaP cells and 10.12 ± 0.86% of DU145 cells. TRAIL was less active against the both prostate cancer cell lines. We confirmed that hormone-sensitivity LNCaP cells and hormone-refractory DU145 prostate cancer cells are resistant to TRAIL.

#### 3.1.3. TRAIL in Combination with EEP

**Figure 3:**
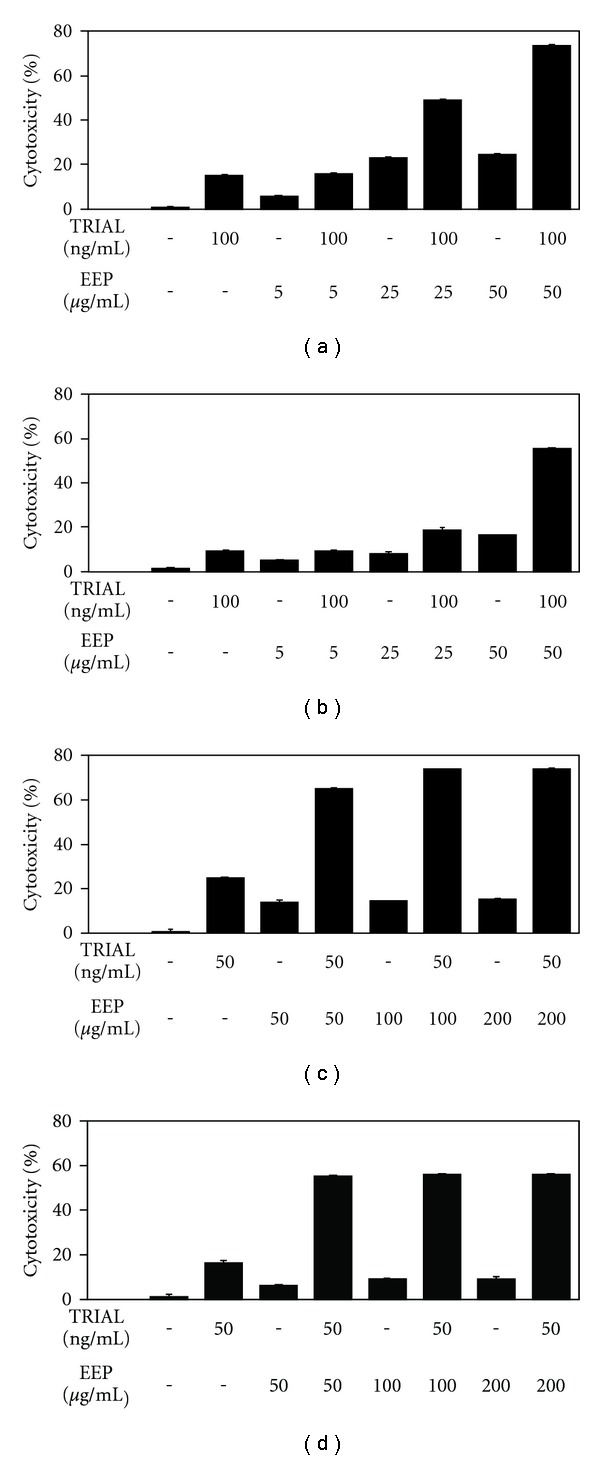
Cytotoxic activity of TRAIL in combination with EEP in prostate cancer cells. The cells: (a) LNCaP and (b) DU145 were incubated for 48 h with TRAIL at the concentrations of 100 ng mL^−1^ and with EEP at concentrations of 5–50 *μ*g mL^−1^. The cancer cells: (c) LNCaP and (d) DU145 were incubated for 48 h with TRAIL at concentrations of 50–200 ng mL^−1^ and EEP at the concentration of 50 *μ*g mL^−1^. The percentage of cell deaths was measured by MTT cytotoxicity assay. The values represent mean ± SD of three independent experiments performed in quadruplicate (*n* = 12). All differences are statistically significant in relation to control (*P* < .05).

**Figure 4:**
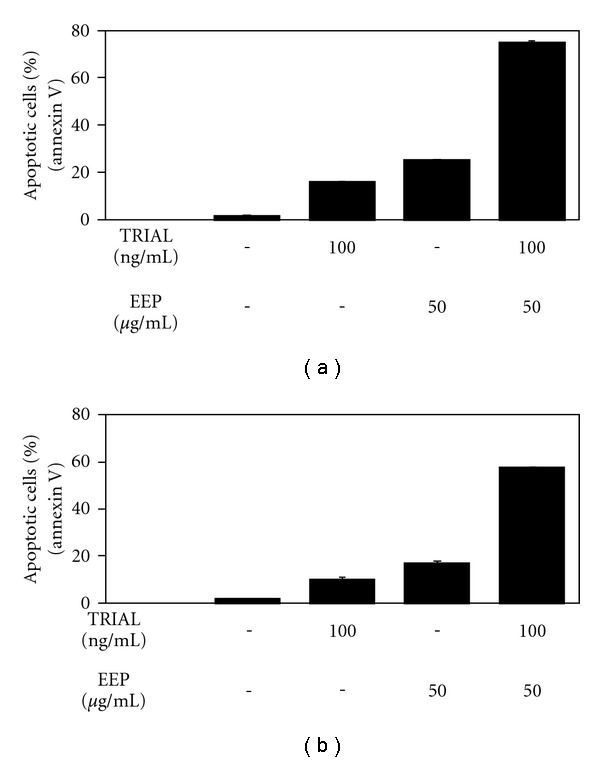
TRAIL induced apoptosis in combination with EEP in prostate cancer cells: (a) LNCaP and (b) DU145. Detection of apoptotic cell death after 48-h cotreatment with TRAIL at the concentration of 100 ng mL^−1^ and EEP the concentration of 50 *μ*g mL^−1^ by annexin V-FITC staining using flow cytometry. The values represent mean ± SD of three independent experiments performed in quadruplicate (*n* = 12). All differences are statistically significant in relation to control (*P* < .05).

We investigated the cytotoxic and apoptotic effects of TRAIL in combination with EEP on prostate cancer cells (Figures 3 and 4). Cotreatment of TRAIL and EEP increased the percentage of cell death on prostate cancer cells, compared to cytotoxicity of TRAIL or EEP alone. The cytotoxicity after 48-h incubation with TRAIL at the concentration of 100 ng mL^−1^, and EEP at the concentration of 50 *μ*g mL^−1^ was 73.70 ± 0.53% for hormone-sensitivity LNCaP cells and 55.76 ± 0.72% for hormone-refractory DU145 cells. Then, we tested the apoptotic effect of TRAIL in combination with EEP on prostate cancer cells. We found that EEP strongly enhanced TRAIL-induced apoptosis in cancer cells. The percentage of apoptotic cells after exposure to 100 ng mL^−1^ TRAIL in combination with 50 *μ*g mL^−1^ EEP increased to 74.94 ± 0.74% for LNCaP cells and to 57.39 ± 0.67% for DU145 cells. Our results indicated that EEP enhanced apoptosis inducing potential of TRAIL in hormone-sensitivity LNCaP and hormone-refractory DU145 prostate cancer cells. Propolis restored sensitivity of prostate cancer cell lines to TRAIL-induced cell death.

The necrotic cell death percentage of prostate cancer cells incubated with TRAIL and/or EEP examined by LDH test was near 0.

**Figure 5:**
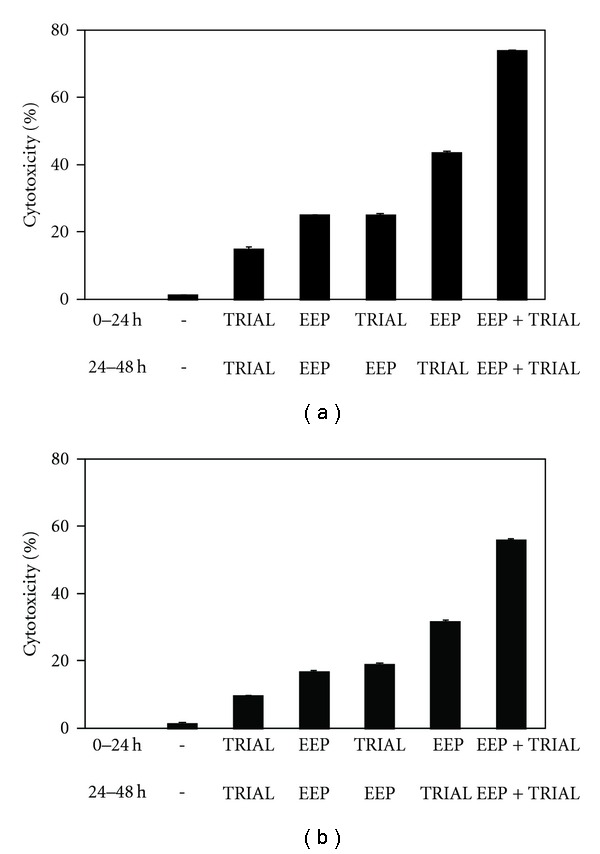
Cytotoxic activity of TRAIL in combination with EEP, after and before exposure to EEP in prostate cancer cells. (a) LNCaP and (b) DU145 cancer cells were as follws: (1) treated with EEP in combination with TRAIL for 48 h; (2) pretreated with EEP for 24 h, followed by TRAIL for another 24 h; and (3) pretreated with TRAIL for 24 h, followed by EEP for another 24 h. The percentage of cell deaths was measured by MTT cytotoxicity assay. The values represent mean ± SD of three independent experiments performed in quadruplicate (*n* = 12). All differences are statistically significant in relation to control (*P* < .05).

The sequence of drug administration is important to obtain maximum therapeutic benefits in combined therapy. We therefore examined whether cotreatment of prostate cancer cells with EEP and TRAIL induced greater apoptosis than the concurrent pretreatment with EEP followed by TRAIL and vice versa (Figure 5). Interestingly, the cotreatment of both prostate cancer cell lines with EEP in combination with TRAIL induced greater apoptosis than concurrent pretreatment or single agent alone. Reverse sequence of treatments: pretreatment with EEP followed by TRAIL or pretreatment with TRAIL followed by EEP resulted in significantly lesser apoptosis than in the cotreatment with EEP and TRAIL.

### 3.2. Cytotoxicity of Studied Agents in Prostate Cancer Cells

#### 3.2.1. Phenolic Compounds Detected in Propolis

**Figure 6:**
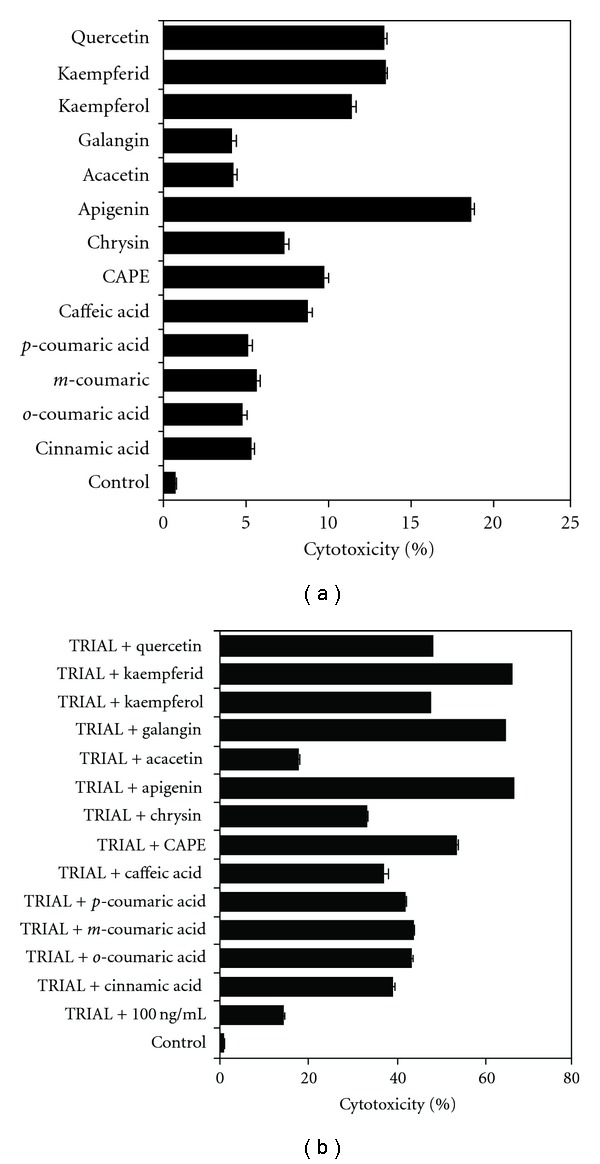
Cytotoxic activity of (a) EEP phenolic components and (b) TRAIL in combination with EEP phenolic components in prostate cancer cells. The LNCaP cells were incubated for 48 h with phenolic compounds at the concentration of 50 *μ*M with or without TRAIL at the concentration of 100 ng mL^−1^. The percentage of cell deaths was measured by MTT cytotoxicity assay. The values represent mean ± SD of three independent experiments performed in quadruplicate (*n* = 12). All differences are statistically significant in relation to control (*P* < .05).

We investigated the cytotoxic effect on LNCaP cells of 13 phenolic components of propolis: cinnamic acid, *o*-coumaric acid, *m*-coumaric acid, *p*-coumaric acid, caffeic acid, CAPE, chrysin, apigenin, acacetin, galangin, kaempferol, kaempferid and quercetin (Figure 6(a)). The strongest cytotoxic activity in cancer cells was demonstrated by apigenin (18.76 ± 0.65% cell death). Kaempferol, kaempferid and quercetin induced few cell deaths (11.42 ± 0.68, 13.44 ± 0.44 and 13.36 ± 0.59%, respectively). The cytotoxicity of other remaining compounds found in propolis in prostate cancer cells was below 10%.

#### 3.2.2. TRAIL in Combination with Phenolic Compounds Detected in Propolis

Cytotoxic effect of TRAIL in combination with phenolic acids or flavonoids in LNCaP cell line measured by MTT assay is shown in Figure 6(b). We tested the effect of a 48-h cotreatment with TRAIL at the concentration of 100 ng mL^−1^ together with 13 phenolic components of propolis at the concentration of 50 *μ*M on cytotoxicity of prostate cancer cells. The phenolic acids and particularly flavonoids restored TRAIL sensitivity in TRAIL-resistant LNCaP cells. In our study, apigenin, kaempferid, galangin and CAPE markedly augmented TRAIL mediated cancer cell deaths (53.51 ± 0.68–66.06 ± 0.62%) and exhibited the strongest cytotoxic effect in combination with TRAIL on LNCaP cells. The other components found in our sample of propolis also increased the percentage of TRAIL-induced cell deaths, compared to cytotoxicity of TRAIL alone, but the cytotoxicity was below 50%.

The necrotic cell death percentage of LNCaP cells incubated with TRAIL and/or phenolic components examined by LDH leakage was near 0.

## 4. Discussion

Epidemiological data support the concept that naturally occurring anticancer agents in the human diet are safe, and nontoxic, and they have long-lasting beneficial effects on human health [12, 21]. The potential target for complementary and alternative medicine (CAM) research has been on the discovery of natural compounds that can be used in prevention against prostate cancer.

The study by Li et al. [22] showed that propolis inhibits cellular proliferation and induces apoptosis in prostate cancer cells. In our investigation, we also observed cytotoxic and apoptotic activities of EEP against hormone-sensitivity LNCaP and hormone-refractory DU145 prostate cancer cells. Beside antitumor effect, immunomodulatory properties of propolis have been recorded. We investigated the interaction between propolis and tumor necrosis factor-related apoptosis inducing ligand on prostate cancer cells. Recombinant human TRAIL used in our study is a soluble protein based on a natural ligand. TRAIL induces programmed death in various cancer cells, *in vitro* and *in vivo* [16]. However, some tumor cells are resistant to TRAIL-mediated cytotoxicity. We and others demonstrated that prostate cancer cell lines, LNCaP and DU145, were resistant to TRAIL-induced apoptosis [17–19].

Our study showed the impact of propolis on the anticancer immune defense. Propolis restores sensitivity of tumor cells to immune effectors mechanisms, such as TRAIL-induced apoptosis in prostate cancer cells. For the first time, our results demonstrated that EEP markedly augmented TRAIL-mediated apoptosis in hormone-sensitivity LNCaP and hormone-refractory DU145 prostate cancer cells. The rapid tumor growth and progression of hormone refractory prostate cancer accounts for most of the morbidity and mortality associated with prostate cancer [1]. The experimental data indicated that propolis is a promising anticancer agent also for the prevention of hormone-refractory prostate cancer.

In the field of CAM, immunomodulation through natural or synthetic substances may be considered as an alternative for the prevention of neoplasm disease. EEP enhances the apoptosis-inducing potential of TRAIL and sensitizes TRAIL-resistant prostate cancer cells. Further investigations will be required to recognize and explain the molecular mechanisms and cellular signaling pathways by which EEP sensitizes cancer cells to TRAIL-induced death. Moreover, due to heterogenous complex composition of propolis, its biological activity is variable. The presence in propolis of so many compounds makes it difficult to know and understand the direct and indirect effects of EEP upon transduction pathway of the signal to TRAIL-mediated apoptosis in cancer cells.

The flavonoids and phenolic components found in propolis are known to affect the apoptosis of prostate cancer cells and may play an important role in cancer chemoprevention [2, 3, 23, 24]. We tested *in vitro* the cytotoxicity of 13 compounds detected in our sample of propolis against prostate cancer. The strongest cytotoxic activity on LNCaP prostate cancer cells was demonstrated by apigenin. Shukla and Gupta [23, 25] reported that apigenin in both *in vitro* and *in vivo* studies induced apoptosis in prostate cancer.

It has been suggested that phenolic compounds isolated from propolis induce activities of the immune system and exert antitumor effects [9–15, 22–25]. To investigate which compounds found in propolis may be responsible for the enhancement of the apoptosis-inducing potential of TRAIL, we tested the cytotoxic effect of its phenolic components in combination with TRAIL on prostate cancer cells. All detected in our EEP sample compounds used in combination with TRAIL increased the percentage of cell deaths compared to cytotoxicity of TRAIL alone. The phenolic acids and particularly flavonoids restored TRAIL sensitivity in TRAIL-resistant LNCaP prostate cancer cells. In our study, apigenin, kaempferid, galangin and CAPE markedly augmented TRAIL mediated cancer cells death and exhibited the strongest cytotoxic effect in combination with TRAIL on LNCaP cells. Apigenin, kaempferid and galangin, the compounds with the most cytotoxic activity with TRAIL, have three hydroxyl groups (positions 5, 7 and 3 or 4′). Every tested flavonoid has hydroxyl groups in fifth and seventh positions. The compounds with only two hydroxyl groups in fifth and seventh positions (chrysin, acacetin), or four (kaempferol) and five hydroxyl groups (quercetin) showed lower cytotoxic activity with TRAIL. The presence of hydroxyl group in position 3 (galangin versus chrysin) decreased activity of galangin, but addition of TRAIL changed this activity. Probably, this activity is dependent on different mechanisms. The position of hydroxyl groups in flavone structure and their number are very important in reaction with reactive oxygen species as well as can influence cytotoxic and apoptotic activities [26, 27].

**Figure 7:**
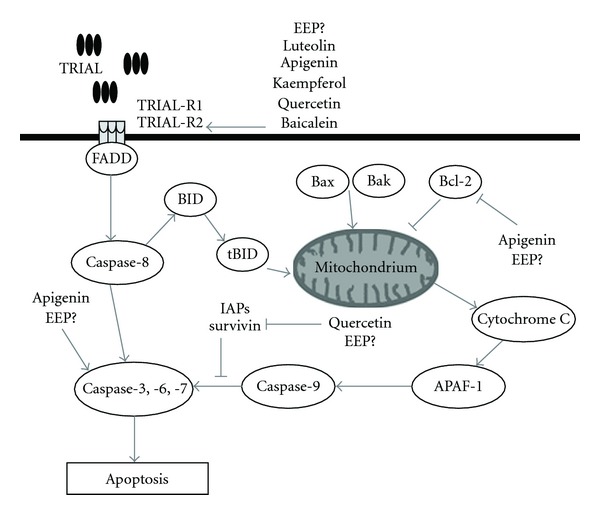
The molecular targets of flavonoids in TRAIL apoptosis pathway and the hypothetic impact of EEP in TRAIL-mediated apoptosis in cancer cell. TRAIL binds to death receptors, TRAIL-R1 and/or TRAIL-R2 and promotes the recruitment of adapter molecule FADD (Fas-associated-death domain) to activate caspase-8, which trigger activation of downstream effector caspases (caspase-3, -6 and -7). Caspase-8 mediated also cleavage of Bid (BH3-interacting domain death agonist). Trucated Bid called tBid translocates to the mitochondria where it interacts with Bax (Bcl-2-associated X protein) and Bak (Bcl-2-agonist/killer), stimulating the release oh cytochrome *c*. Antiapoptotic members of Bcl-2 family could inhibit cytochrome *c* release. Cytochrome *c* liberated from the mitochondria then binds to the adaptor protein APAF-1 (apoptotic protease-activating factor-1) and procaspase-9, forming the apoptosome and activating caspase-9, which in turn activates executioner caspases (caspase-3, -6 and -7) leading to cell death. Caspase-9 activity is controlled by IAPs (inhibitor of apoptosis protein) and survivin. Luteolin, apigenin, kaempferol, quercetin and baicalein increase expression of death receptor TRAIL-R2. Apigenin activate executioner caspases and block antiapoptotic Bcl-2 action. Quercetin promotes apoptosis by inhibition of survivin.

In study *in vitro* on HeLa cell line, we confirmed that EEP sensitize cancer cells to TRAIL-induced apoptosis and two components identified in propolis, apigenin and CAPE, were the most potent agents inducing cell death in combination with TRAIL in HeLa cells [20]. A similar study with flavonoids (Figure7) showed that luteolin, apigenin, kaempferol, baicalein and quercetin synergistically induced apoptosis with TRAIL in human malignant tumor cells [18, 28–33]. Horinaka et al. [28] reported that luteolin increased TRAIL-induced apoptosis in HeLa cells through upregulation of death receptor TRAIL-R2. In other investigation, they also showed the enhanced apoptosis-inducing potential of TRAIL in prostate cancer cell line DU145, leukemic cell line Jurkat, and colon cancer cell line DLD1. The combined use of apigenin and TRAIL caused Bcl-2-interacting domain cleavage, activation of caspases, and increased expression of TRAIL-R2 [18]. Yoshida et al. [29] stated that TRAIL-R2 upregulation by kaempferol augments TRAIL action in colon cancer cells [29]. Chen et al. [30] showed that suppression of survivin and induction of TRAIL-R2 by quercetin contribute to sensitization of lung cancer cells to TRAIL-induced cytotoxicity. Kim et al. [31] examined the molecular mechanisms by which quercetin augments TRAIL-mediated apoptotic death in prostate cancer cells and confirmed the ability of quercetin to downregulate survivin expression. Taniguchi et al. [32] indicated that baicalein increases TRAIL-R2 expression and overcomes TRAIL resistance in prostate cancer cells.

We demonstrated for the first time that kaempferid, galangin and CAPE enhance the cytotoxic potential of TRAIL in prostate cancer cells. Those polyphenols, beside apigenin, equally firmly sensitize TRAIL-resistant LNCaP cells. The previous study suggested that flavonoids increase expression of TRAIL-R2 [18, 28–30, 32]. We hypothesize that propolis, as one of the richest sources of flavonoids, such as apigenin, kaempferol, kaempferid, galangin, quercetin and CAPE, can influence the expression of death receptor TRAIL-R2, inhibition of antiapoptotic protein (Bcl-2, survivin), or activation of caspases (Figure 7).

We showed that EEP and its phenolic components *in vitro* augmented TRAIL mediated cell death in prostate cancer, but further study will be required to examine the molecular mechanisms by which EEP and its compounds act on cellular signaling pathways and sensitize prostate cancer cells to TRAIL-induced apoptosis. Our findings suggest that the modulation of TRAIL apoptosis pathway may have a significant potential for prostate cancer chemoprevention, and the overcome of TRAIL-resistance by propolis and its phenolic components may be one of the mechanisms responsible for their cancer preventive effects. The obtained results confirmed the significance of EEP and its components in chemoprevention of prostate cancer cells. EEP as a dietary supplement may be useful in chemoprevention agent against prostate cancer.

## Funding

Research Grant 2-164/08 from Medical University of Silesia in Katowice, Poland.

## Figures and Tables

**Table 1:** Chemical structure of the phenolic compounds used in this study.